# “Magic Bullet”: Eccentric Macular Hole as a Complication from Dexamethasone Implant Insertion

**DOI:** 10.1155/2016/1706234

**Published:** 2016-10-05

**Authors:** Logan Christensen, Riley Sanders, Jeffrey Olson

**Affiliations:** Department of Ophthalmology, University of Colorado, 1674 Aurora Ct., Aurora, CO 80045, USA

## Abstract

*Introduction*. Intravitreal drug injections and implants are generally safe but do carry some risk, from both the procedure itself and adverse effects of the medications. We report a case of an eccentric macular hole after dexamethasone implant (Ozurdex®) administration.* Ex vitro* force testing was performed to evaluate dexamethasone implant injection force.* Methods*. Five dexamethasone implant (Ozurdex) applicators were placed 16 mm from a force plate and the force of the injected dexamethasone pellet was recorded in Newtons. Four dexamethasone implant applicators were placed 16 mm from a force plate in a basic saline solution and the force of the pellet was recorded.* Results*. Average maximum force in air was 0.77 N and 0.024 N in a basic saline solution (BSS).* Conclusion*. We present a case report of an eccentric macular hole after dexamethasone implant administration. We hypothesize a mechanical injury to the retina during insertion caused the macular hole. Force testing done in air demonstrated sufficient force from the pellet injection to cause retinal damage though injections done in BSS showed reduced forces.

## 1. Introduction

Ozurdex is an extended-release dexamethasone implant injected intravitreally for the treatment of noninfectious uveitis and macular edema. In contrast to other delivery systems, increased steroid levels can last on the order of months as compared to weeks.

As with other steroid therapies, elevated intraocular pressure and cataract progression are the most common adverse events. The implant, however, requires the injection of a solid pellet into the eye which carries its own procedural risks, including macular holes [1].

In the following case, a patient is presented who developed an eccentric macular hole following dexamethasone pellet implantation. Experimental data from a laboratory study was collected to measure the mechanical force associated with implant administration.

## 2. Case History

**Figure 1:**
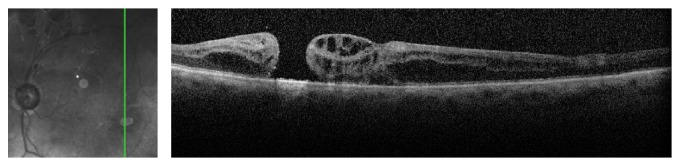
OCT taken after implant insertion showing inferotemporal eccentric macular hole.

The patient is a 76-year-old Russian-American with a history of bilateral primary open angle glaucoma status after bilateral trabeculectomy and on topical timolol daily bilaterally. The patient was pseudophakic bilaterally and also had a history of bilateral Fuch's dystrophy with corneal edema for which he had received a left Descemet's stripping automated endothelial keratoplasty (DSAEK). The patient developed left macular edema of unknown origin and was treated with intravitreal bevacizumab ×2. After poor response to intravitreal anti-VEGF injections, the patient traveled to Europe where he received a dexamethasone implant for the recalcitrant macular edema into the left eye. Prior to implant insertion, fundus exam was only remarkable for macular edema. In the exam following implant insertion, an eccentric macular hole was noted with an intact posterior vitreous face. Postinjection Optical Coherence Tomography (OCT) can be seen in Figure 1. The hole was not causing visual symptoms and has been monitored without change since that time.

## 3. Methods

Nine dexamethasone implant samples (Allergan Pharmaceuticals) were used for testing. The tip of the applicator was placed 16 mm, the estimated distance of travel during an injection administration [2], from a force plate connected to a force transducer (MLT 1030 wide range force transducer, ADInstruments). The actuator of the implant device was pushed at by a constant motion of medium speed until the implant was expelled from the device. The deflection of the force plate from the impact of the implant was recorded by the computer software and calibrated to record in Newtons (N).

**Figure 2:**
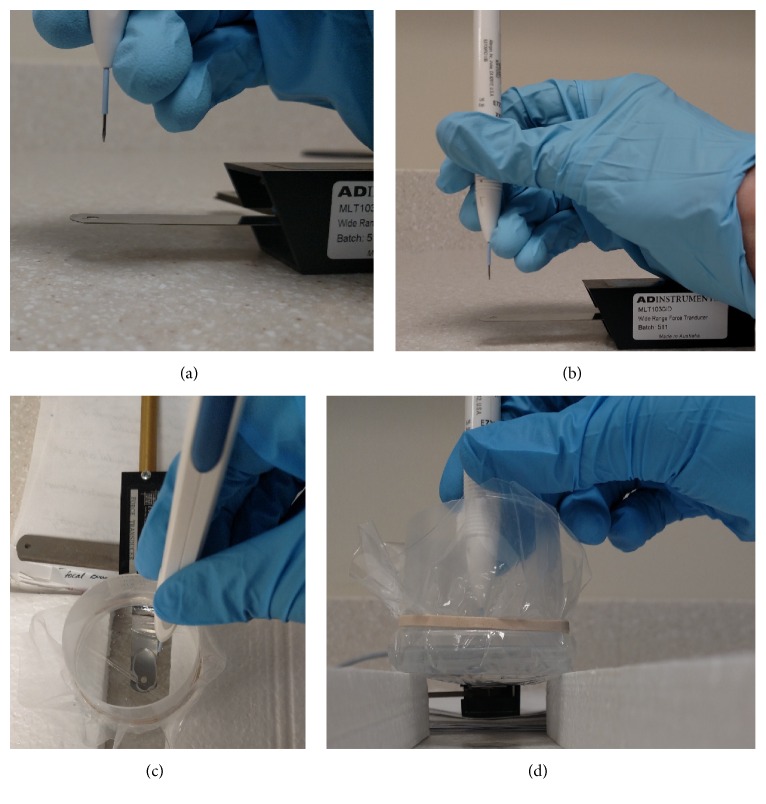
Experimental setup. (a) and (b) show setup for air. Ozurdex applicator held 16 mm from the force plate. Force plate readings measured in Newtons. (c) and (d) demonstrate setup for basic saline solution (BSS). Container with thin plastic bottom placed so that it made contact with force plate. Ozurdex applicator held 16 mm from the force plate. Force plate readings measured in Newtons.

A specimen cup with the bottom removed was covered with a thin plastic film. The force blade was placed at a level so that the film barely made contact with the force blade. The specimen cup was filled with basic saline solution (BSS) to a depth of 16 millimeters. The applicator was placed perpendicularly and fully submersed in the BSS. The implant was injected onto the edge of the force blade. See Figure 2 for experimental setup.

**Figure 3:**
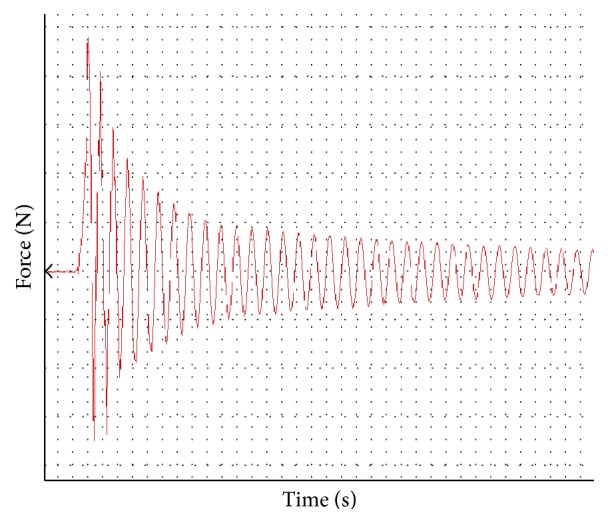
Ozurdex injection data. *x*-axis is time in seconds. *y*-axis is force in Newtons. Maximum force was measured from baseline to maximum value in the *y*-axis.

Readings were analyzed using the ForceLab software version 8.0 (ADInstruments). Maximum force (N) was recorded as the difference between the maximum measured force and baseline. See Figure 3 for representative data.

## 4. Results

The average force reading for the implant administration in air was 0.77 N with a standard deviation of 0.26. The average force reading of the implant administration in basic saline solution was 0.024 N with a standard deviation of 0.031.

## 5. Discussion

Vitreous traction from the implant procedure has been proposed as a mechanism creating central macular holes [1, 3]. In the case presented here, the postprocedure macular hole was eccentric, making a vitreous traction mechanism less likely. Additionally, the patient had not undergone posterior segment surgery to explain the macular hole development. Direct mechanical damage from impact of the dexamethasone implant onto the retina is thought to be improbable based on kinematic studies with a high-speed camera [2] and the safety profile in the CHAMPLAIN study group for dexamethasone implant® in vitrectomized eyes [4]. Kinematic studies indicate that a direct impact of an dexamethasone implant pellet onto the retina is unlikely and thought not to have enough energy to produce significant damage [2]. Although there is some controversy regarding the correlation between the speed of actuator depression and implant exit velocity, exit velocities appear to be consistent across studies [2, 5]. For the purposes of this study, the exit velocity was assumed to be equal among all tested implant injections.

The force testing presented here shows a maximum impact force of 0.77 N when fired from 16 mm in air. Previous experiments have shown an ability to cause retinal damage with a force between 0.1 and 0.2 N [6]. Although measured impact force was less in the BSS solution, the air results suggest that there could hypothetically be enough force to cause retinal damage. This “magic bullet” hypothesis suggests that although the average impact force in BSS or vitreous is below the threshold for retinal damage, a dexamethasone implant is expelled from the applicator with enough force to cause retinal damage. Additionally, it is difficult to estimate the effect of chronic retinal pathology on susceptibility to injury. Perhaps a patient with existing retinal disease would be predisposed to injury from a lesser insult. In the case of this patient, we are lacking any other explanation other than a direct mechanical impact of the dexamethasone implant dexamethasone pellet onto the retina creating a macular hole.

It is possible that this patient suffered an exceedingly rare complication from dexamethasone implant insertion. The pellet may have ejected from the insertion device and retained enough kinetic energy through the posterior segment to impact the retina. Rather than tumbling, it may have maintained a linear trajectory. Force testing in air media with dexamethasone implant applicators showed a maximum impact force of 0.77 N, which is more than strong enough to cause such damage though unlikely given the measured decreased force in other media.
